# Survey of Gastrointestinal Parasites of Farmed Pigs in Ghana

**DOI:** 10.1002/vms3.71131

**Published:** 2026-08-06

**Authors:** Antoinette V. Keleve, Sylvia Afriyie Squire, Doreen Dela Owusu‐Ntumy, Dennis Adusei, Linda Ama Owusuaa Amoah

**Affiliations:** ^1^ Council for Scientific and Industrial Research – Animal Research Institute Accra Ghana; ^2^ CSIR College of Science and Technology, Council for Scientific and Industrial Research – Animal Research Institute Accra Ghana; ^3^ Hawa Memorial Saviour Hospital Osiem Eastern Region Ghana

**Keywords:** coproscopy, *Cryptosporidium;* gastrointestinal parasites, Ghana, pigs, prevalence

## Abstract

**Background:**

Pig farming is an important source of income in Ghana, but gastrointestinal parasitism remains a constraint to productivity. Information on parasite occurrence in pig production systems is limited.

**Objectives:**

The objective of this study is to determine prevalence and infection intensity of gastrointestinal parasites in pigs in Kpone Katamanso Municipality, Ghana, using complementary coproscopic methods, with polymerase chain reaction (PCR) used to supplement *Cryptosporidium* detection.

**Methods:**

A cross‐sectional study examined faecal samples from 142 pigs in Kpone Katamanso. Gastrointestinal parasite eggs, larvae, and oocysts were detected using the formol–ether concentration, modified McMaster, and modified Ziehl–Neelsen (MZN) techniques. A subset of 92 samples, selected independently of their MZN results, was analysed for *Cryptosporidium* spp. by nested PCR targeting the 18S rRNA gene.

**Results:**

Overall gastrointestinal parasite prevalence by coproscopy was 67.6% [96/142]. Five parasites were identified: *Eimeria* spp. (34.5%), strongyles (27.5%), *Cryptosporidium* spp. (21.8%), *Balantioides* spp. (20.4%), and *Trichuris* spp. (10.6%). All farms had at least one positive pig, with farm‐level prevalence ranging from 28.6% to 100%. Combining coproscopic techniques improved parasite detection. Among the 92 samples analysed by PCR, *Cryptosporidium* prevalence was 29.3% (27/92) and 45.7% (42/92) when PCR and MZN results were combined. Infection intensity was generally moderate to heavy.

**Conclusion:**

Gastrointestinal parasites were highly prevalent in pigs sampled in Kpone Katamanso. Complementary coproscopic techniques improved parasite detection, while PCR complemented detection of *Cryptosporidium*. These findings provide baseline data for parasite surveillance and diagnostic method selection. Species‐level identification is needed to determine the zoonotic significance.

## Graphical abstract

Gastrointestinal parasites were common in pigs sampled from Kpone Katamanso Municipality, Ghana, with an overall prevalence of 67.6%. Complementary formol–ether concentration, modified McMaster, and modified Ziehl–Neelsen techniques improved parasite detection, while polymerase chain reaction identified additional *Cryptosporidium* infections within the analysed subset. These findings provide baseline epidemiological evidence to support parasite surveillance, diagnostic method selection, and future species‐level molecular investigations.

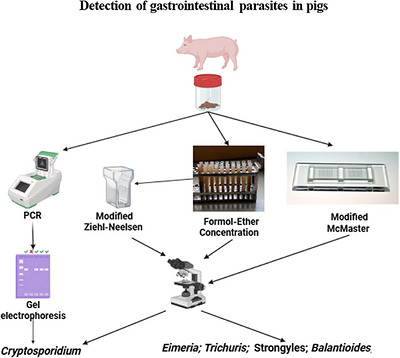

## 1 Introduction

The global pig production industry is experiencing significant growth, driven by increasing demand for pork (Omoruyi and Agbinone 2020). In sub‐Saharan Africa, Latin America, and Southeast Asia, pig farming is a vital source of income and employment, providing an essential source of animal protein for consumption (Roesel et al. 2017; Adesehinwa et al. 2024; Weka et al. 2021). Despite the economic importance of pig production, the industry faces significant challenges, including diseases and parasitic infections (Ngwili et al. 2022). Gastrointestinal parasites, in particular, pose a major threat to pig production, affecting productivity, profitability, and meat quality (Njoga et al. 2018; Okoli et al. 2018; Symeonidou et al. 2020), while some species also pose a risk to human health through zoonotic transmission.

Pig production in Ghana is characterised by diverse systems, ranging from traditional extensive systems with minimal shelter and free‐range movement to intensive systems with full confinement (Banson et al. 2014; Adjei et al. 2015). The majority of farms are small‐scale, relying on kitchen leftovers to feed the pigs and occasionally supplemented with concentrates or agro‐industrial byproducts (Aryee et al. 2019). Despite its potential, Ghana's pig production industry faces significant challenges, including a persistently high prevalence of gastrointestinal parasitic infections, with reported rates of over 80% in certain regions (Osei et al. 2023; Addy et al. 2023).

Current knowledge of gastrointestinal parasite epidemiology in Ghanaian pigs remains limited, hindering the development of evidence‐based control measures. Existing studies have primarily focused on the Ashanti Region and northern Ghana, employing coproscopy (Permin et al. 1999; Atawalna et al. 2016; Larbi et al. 2022; Osei et al. 2023; Addy et al. 2023). To address this knowledge gap, further research is necessary to investigate the epidemiology of gastrointestinal parasites in other agroecological zones, considering the impact of varying climatic conditions and husbandry practices on parasite prevalence and distribution.

While coproscopy and microscopic examination of stool samples for intestinal parasites offer advantages, such as affordability, ease of performance in resource‐limited settings, and detection of clinically significant parasites, they also have limitations. These limitations include the need for specialised technical expertise, lengthy processing times, and the potential for false negatives, particularly when parasites have low egg counts or irregular shedding patterns (Garcia and Shimizu 1997; Dryden et al. 2005; Zajac and Conboy 2012; Taylor et al. 2015). To improve detection rates, molecular techniques like polymerase chain reaction (PCR) assays can be employed. However, these methods also have limitations, including high costs, specialised equipment requirements, and potential false negatives due to inhibition or poor DNA extraction (Thompson et al. 2017). Consequently, there is a need to identify practical diagnostic approaches that maximise parasite detection while remaining feasible under field and routine laboratory conditions, particularly in resource‐limited settings. This study therefore employed complementary coproscopic techniques to determine the prevalence and intensity of gastrointestinal parasites in pigs in Kpong Katamanso, Ghana, while nested PCR was used to supplement the detection of *Cryptosporidium* spp. The study also compared the parasite detection achieved by the different coproscopic techniques.

By generating baseline epidemiological data and comparing complementary diagnostic approaches, this study contributes evidence to support parasite surveillance and informed selection of diagnostic methods for gastrointestinal parasites in pigs. The findings provide a basis for improving parasite control programmes in Ghana and are relevant to pig production systems in other low‐ and middle‐income countries where gastrointestinal parasitism remains endemic and routine diagnosis relies largely on conventional parasitological methods.

## 2 Materials and Methods

### 2.1 Study Area

This study was conducted in the Kpone Katamanso Municipality, which is located in the Eastern part of the Greater Accra Region. It is about a 38‐kilometre drive from the capital of Ghana, Accra, with mostly urban settlements. The district is located in Ghana's coastal savannah zone, characterised by a dry equatorial climate with annual rainfall of 730–790 mm. The area experiences two rainy seasons, with temperatures ranging from 25°C to 35°C throughout the year. The vegetation consists of shrubs and grasslands (GSS, 2014). According to the 2021 Population and Housing Census, the population of Kpone Katamanso is 417,334, representing 7.6 percent of the region's total population (GSS, 2021). Although the report on the 2021 population and household census does not indicate the number of animals kept in the municipality, the 2010 Population and Housing Census reported 54,495 farmed animals kept by 1359 people, the majority of which were chicken (GSS, 2014). The report indicated a pig population of 2970, with an average of 66 animals per keeper.

### 2.2 Study Design and Sample Collection

#### 2.2.1 Sampling Strategy

This cross‐sectional study was conducted in the KKMA between June and September 2023. Because no complete census of pig farms was available in Kpone Katamanso, a non‐probability sampling approach was employed. Convenience sampling was used to identify initial farms from lists maintained by Kpone Katamanso livestock officers. Additional farms were recruited through snowball sampling, whereby participating farmers referred other pig producers. The number of farms was not predetermined; farms were visited sequentially until the cumulative animal target was achieved.

#### 2.2.2 Pig Selection and Sample Collection

Within each farm, pigs aged approximately 2–12 months were selected as encountered, with a maximum of 20 pigs sampled per farm. On farms with fewer than 20 eligible pigs, all were included. Sampling continued until the cumulative target of 142 pigs was reached, which occurred on the 13th farm. Faecal samples were collected into labelled containers, stored on ice, and transported to the laboratory for analysis. Faecal samples were collected via rectal palpation into labelled containers, stored on ice, and transported to the laboratory for analysis. To minimise cross‐contamination, separate clean disposable gloves were used for each sample.

#### 2.2.3 Questionnaire Survey and Data Collection

Questionnaires were distributed to all 13 participating farms to collect information on farm management practices. The structured questionnaire addressed farmer demographics, farm management practices, and the history of deworming of farm animals. Only seven farms returned completed questionnaires, and some questions remained unanswered due to inconsistent record‐keeping. Farms with incomplete or missing responses were excluded from the analysis. Consequently, treatment and management variables were not included in the final statistical analysis because the low response rate yielded insufficient data for meaningful analysis.

#### 2.2.4 Sample Size Determination

For planning purposes, the target sample size of 142 pigs was estimated using Cochran's formula (Cochran 1977) at a 95% confidence interval and 5% margin of error, based on a previously reported prevalence of 91.1% for gastrointestinal parasites in pigs from Tolon and Kumbungu Districts, Ghana (Addy et al. 2023). A 13% contingency was included to account for potential sample losses. Due to the use of non‐probability sampling at the farm and pig levels, assumptions of Cochran's formula could not be satisfied.

#### 2.2.5 Limitations and Generalisation

Because no complete census of pig farms was available, convenience and snowball sampling were used to identify farms. While this approach ensured feasibility, it introduces potential selection bias and limits representativeness. The prevalence estimates should therefore be interpreted with caution.

### 2.3 Coprological Analysis

Each stool sample underwent processing using sedimentation (formol–ether concentration [FEC]) and flotation (modified McMaster [MM] and modified Ziehl–Neelsen [MZN]) techniques. The compound microscope was utilised to examine suspensions of faecal pellets and faecal smears, using ×10, ×40, and ×100 objectives. Afterwards, the number of parasite eggs or oocysts per gram of faeces (EPG/OPG) was recorded. Eggs, larvae, and oocysts lacking clear distinguishing features, including those with *Taenia*‐like features, were excluded to avoid misidentification and reporting of spurious passage. All slides were examined independently by two trained technicians. Discrepant results were re‐examined jointly until consensus was reached to minimise reader bias.

#### 2.3.1 Formalin–Ether Concentration

Briefly, 1 g of faecal sample was emulsified with 4 mL of 10% formol water and filtered into a centrifuge tube for FEC. Diethyl ether was added, shaken, and centrifuged at 3000 rpm for 1 min. The supernatant was discarded, and the sediment was mixed and applied to a grease‐free microscope slide. Lugol's iodine stain was used for microscopic examination at ×10 and ×40 objectives. Parasites were identified based on morphological structures.

#### 2.3.2 Modified McMaster Technique

The improved MM method was used to detect and identify intestinal parasitic oocysts, eggs, and larvae as described by Taylor et al. (2015). Briefly, 3 g of faecal sample was macerated with 42 mL of water and filtered through a fine‐meshed sieve. The filtrate was centrifuged at 1500 rpm for 3 min in a 15 mL test tube, and the supernatant was discarded. The deposit was resuspended with saturated salt (NaCl) solution to the previous volume. The resulting suspension (0.15 mL) was transferred to a McMaster chamber and allowed to settle for about 3 min before microscopic examination for parasite eggs. The detection limit for the MM technique was 50 EPG/OPG.

#### 2.3.3 Modified Ziehl–Neelsen Technique

Smears of concentrated faecal samples using the MM technique were prepared on glass slides for *Cryptosporidium* detection using an MZN staining procedure. The smears were air‐dried, fixed with methanol for 5 min, and then stained with strong carbol‐fuchsin for 15 min. After rinsing with tap water, the smears were differentiated with acid alcohol for 15 s and counterstained with 0.4% malachite green for 30 s. Finally, the stained slides were air‐dried and examined under oil immersion at ×1000 magnification to identify *Cryptosporidium* parasites as previously described (Taylor et al. 2015).

### 2.4 Molecular Analysis

#### 2.4.1 Isolation of DNA

From the 142 faecal samples, a subset of 92 was randomly selected for DNA extraction and nested PCR. Selection was independent of coproscopy results to minimise verification bias. The subset comprised 22 samples positive and 70 samples negative by microscopy.

DNA extraction was performed on 250 mg of each faecal sample using the PowerSoil DNA purification Kit (MolBio, Carlsbad, California), with modifications as outlined by Yang et al. (2015). Into each PowerBead Tube (MO‐BIO Laboratories Inc.), 0.25 g of faecal matter was loaded and vortexed to homogenise the stool samples with the lysis buffer. To facilitate efficient oocyst lysis, faecal samples underwent four cycles of freeze–thawing in liquid nitrogen, followed by immersion in boiling water, prior to DNA extraction using the manufacturer's standard protocol. The final elution of DNA was with 50 µL elution buffer. Blank controls were incorporated during DNA extraction to monitor contamination.

#### 2.4.2 Amplification of *Cryptosporidium* by Nested PCR

All the DNA samples were subjected to nested PCR using primers SHP1, SHP2, SHP3, and SSU‐R3 from Silva et al. (2013). The primer pairs SPH1 (ACCTATCAgCTTTAgACggTAgggTAT) and SHP2 (TTCTCATAAggTgCTgAAggAgTAAgg), followed by SHP3 (ACAgggAggTAgTgACAAgAAATAACA) and SSU‐R3 (AAggAgTAAggAACAACCTCCA), targeted the 18S rRNA gene. The first amplification was done in a reaction volume of 25 µL containing 5 µL DNA template, 5 µL 5X Go Taq buffer, 0.5 µL dNTPs (10 mM), 1 µL MgCl_2_ (25 mM), Go Taq DNA polymerase (5u/µL), 1 µL each of primers (10 µM), and nuclease‐free water. The reaction was taken through a denaturation step at 94°C for 3 min and then 35 cycles of 94°C for 30 s, 58°C for 30 s, and 72°C for 1 min. A final extension at 72°C for 7 min ended the reaction. The same reaction conditions for the first PCR were used for the second, but with primers SHP3 and SSU‐R3. The cycling conditions were also the same, except for the annealing temperature, which was 56°C in the second PCR. Both PCRs were done with Promega (Madison, WI, USA) PCR reagents. Blank controls were included during PCR to monitor for contamination. DNA from confirmed *Cryptosporidium* isolates from the Department of Animal Biology and Conservation Science, University of Ghana, was used as a positive control. Amplicons were resolved on a 1% agarose gel stained with ethidium bromide. Positive samples exhibited banding patterns equal to the positive control on agarose gel.

### 2.5 Statistical Analysis

The data collected were entered into Microsoft Excel and exported to SPSS version 27 (IBM Corp, Armonk, NY, USA) for analysis, with a detection limit of 50 EPG/OPG. Prevalence was calculated as the proportion of positive pigs among those examined and expressed as percentages with Wilson score 95% confidence intervals. Overall prevalence, parasite‐specific prevalence, farm‐level prevalence, and prevalence of single and mixed infections were calculated using coproscopy results only. PCR results were excluded from all prevalence analyses except the targeted comparison of MZN staining and nested PCR for *Cryptosporidium* spp.

Because farms were selected using convenience and snowball sampling and pigs were selected opportunistically within farms, prevalence estimates were interpreted as descriptive of the sampled population and not as unbiased estimates for all pigs in Kpone Katamanso or Ghana. Differences in farm‐level prevalence were therefore interpreted cautiously.

Agreement between comparable diagnostic methods was assessed using Cohen's kappa, observed agreement, and expected agreement. Where paired results were available, McNemar's test was used to compare discordant paired proportions. Agreement was interpreted using the Landis and Koch classification. The term “comparative parasite detection” was used instead of “diagnostic accuracy” because no universal reference standard was used.

EPG/OPG values were summarised using range, mean, and standard deviation. Infection intensity was classified using a modified Hansen and Perry framework as low (<200 EPG/OPG), moderate (200–800 EPG/OPG), and high (>800 EPG/OPG). The modification involved application of uniform thresholds to all parasites rather than parasite‐specific values, appropriate for this epidemiological survey of mixed infections. These categories were interpreted as descriptive measures of egg/oocyst shedding, not direct clinical severity.

## 3 Results

### 3.1 Demographic Characteristics of Pigs

All 13 farms in this study kept their pigs under the intensive system by housing them in simple pig sties with concrete floors and sheds. The pigs were provided with formulated feed and/or kitchen and fruit and vegetable wastes as well as water. All the farmers had some understanding of intestinal parasites and thus routinely administered anthelmintics to their pigs every 3–12 months or when the pigs were unwell.

### 3.2 Overall Prevalence of Gastrointestinal Parasites in Pigs Using Coproscopy (*N* = 142)

Examination of 142 pig samples using a combination of FEC, MM, and MZN staining revealed an overall prevalence of 67.6% (95% CI: 59.5−74.8) for at least one gastrointestinal tract parasite. Among the 96 positives, 48 were single infections (one parasite only), while the remaining were mixed infections (more than one parasite), as shown in Table 1.

**TABLE 1:** Distribution of infection categorised by number of gastrointestinal parasites detected.

Infection category	Number positive	Prevalence (%)	95% CI
Single	48	33.8	26.5–41.9
Mixed	48	33.8	26.5–41.9
Total (single + mixed)	142	67.6	59.5–74.8

*Note*: *Cryptosporidium* spp. positivity excludes PCR dataset.

*Eimeria* spp. was the most prevalent parasite detected in 49 of 142 pigs (34.5%; 95% CI: 27.2–42.6), followed by strongyles (27.5%; 95% CI: 20.8–35.3), *Cryptosporidium* spp. (21.8%; 95% CI: 15.8–29.3), *Balantioides* spp. (20.4%; 95% CI: 14.6–27.8), and *Trichuris* spp. (10.6%; 95% CI: 6.5–16.7), as indicated in Table 2. However, confidence intervals overlapped, suggesting minimal differences in prevalence among the various parasites. Therefore, these differences should be interpreted descriptively. These prevalence estimates exclude *Cryptosporidium* by PCR methods.

**TABLE 2:** Gastrointestinal parasites detected using a combination of formol ether concentration, modified McMaster, and modified Ziehl–Neelsen staining techniques (*N* = 142).

Taxonomic group	Parasites detected	Number positive	Prevalence (%)	95% Confidence interval
Nematoda	*Trichuris* spp.	15	10.6	6.5–16.7
	Strongyles	39	27.5	20.8–35.3
Ciliophora	*Balantioides* spp.	29	20.4	14.6–27.8
Apicomplexa	*Eimeria* spp.	49	34.5	27.2–42.6
	*Cryptosporidium* spp.	31	21.8	15.8–29.3

*Note*: *Cryptosporidium* spp. exclude PCR dataset.

### 3.3 Farm‐Level Prevalence of Gastrointestinal Parasites

All 13 farms in the study had at least one gastrointestinal parasite present, resulting in a 100% herd prevalence using a combination of FEC, MM, and MZN staining. At the farm level, prevalence ranged from 28.6% (95% CI: 8.2−64.1) on farm F3 to 100% (95% CI: 77.2–100.0) on farm F11. Comparing prevalence between farms, three farms (F1, F3, and F10) had lower parasite prevalences than the remaining 10 farms. Based on the number of parasites detected, four of the farms (F1, F3, F5 and F10) had only single infections detected, one farm (F10) had only mixed infections detected, while the remaining eight farms had both single and mixed parasite infections detected (Table 3).

**TABLE 3:** Farm‐level prevalence and distribution of gastrointestinal parasites in naturally infected pigs categorised by the number of parasites detected across 13 farms.

		Number positive (%, 95% CI)		
Farm	Number of pigs sampled	Single infection	Mixed infections	Total
F1	20	6 (6.0; 14.5‐51.9)	0 (0.0; 0.0–16.1)	6 (30.0; 14.5–51.9)
F2	20	3 (15.2; 5.2–36.0)	11 (55.0; 34.2–74.2)	14 (70.0; 48.1–85.5)
F3	7	2 (28.6; 8.2–64.1)	0 (0.0; 0.0–35.4)	2 (28.6; 8.2–64.1)
F4	8	3 (37.5; 13.7–69.4)	2 (25.0; 7.1–59.1)	5 (62.5; 30.6–86.3)
F5	8	6 (75.0; 40.9–92.9)	0 (0.0; 0.0–32.4)	6 (75.0; 40.9–92.9)
F6	9	3 (33.3; 12.1–64.6)	6 (66.7; 35.4–87.9)	9 (100.0; 70.1–100.0)
F7	14	7 (50.0; 26.8–73.2)	2 (14.3; 4.0–39.9)	9 (64.3; 38.8–83.7)
F8	10	2 (20.0; 5.7–51.0)	8 (80.0; 49.0–94.3)	10 (100.0; 72.2–100.0)
F9	10	4 (40.0; 16.8–68.7)	4 (40.0; 16.8–68.7)	8 (80.0; 49.0–94.3)
F10	8	3 (37.5; 13.7–69.4)	0 (0.0; 0.0–32.4)	3 (37.5; 13.7–69.4)
F11	13	0 (0.0; 0.0–22.8)	13 (100.0; 77.2–100.0)	13 (100.0; 77.2–100.0)
F12	6	3 (50.0; 18.8–81.2)	1 (16.7; 3.0–56.4)	4 (66.7; 30.0–90.3)
F13	9	6 (66.7; 35.4–87.9)	1 (11.1; 2.0–43.5)	7 (77.8; 45.3–93.7)
*Total*	*142*	*48 (50.0; 40.2–59.8)*	*48 (50.0, 40.2–59.8*	*96 (67.0;* 59.5‐74.8)

*Note*: *n* = number of pigs sampled from farm; 95% CI = Wilson 95% confidence interval; *Cryptosporidium* spp. exclude PCR dataset.

Farm‐level parasite prevalence varied across the 13 sampled farms (Table 4). *Eimeria* spp. were most frequent in F6, F8, F7, F5, and F11, while strongyles were most frequent in F11, F2 and F6. *Cryptosporidium* spp. detected by MZN staining was most frequent in F8, F9, and F4. PCR results were excluded from these analyses. *Balantioides* spp. was concentrated mainly in F11, F12, and F13, while *Trichuris* spp. was detected at lower prevalence across farms. However, several farm‐level confidence intervals were wide because of small numbers of pigs sampled per farm. These differences should therefore be interpreted descriptively rather than as evidence of causal farm‐level effects, particularly because farms were selected using convenience and snowball sampling.

**TABLE 4:** Farm‐level parasite prevalence categorised by specific parasite detected across 13 farms.

Farm	*n*	*Trichuris* spp. (%; 95% CI)	Strongyles (%; 95% CI)	*Balantioides* spp. (%; 95% CI)	*Eimeria* spp. (%; 95% CI)	*Cryptosporidium* spp. (%; 95% CI)
F1	20	0 (0.0; 0.0–16.1)	0 (0.0; 0.0–16.1)	0 (0.0; 0.0–16.1)	0 (0.0; 0.0–16.1)	6 (30.0; 14.5–51.9)
F2	20	6 (30.0; 14.5–51.9)	14 (70.0; 48.1–85.5)	3 (15.0; 5.2–36.0)	3 (15.0; 5.2–36.0)	1 (5.0; 0.9–23.6)
F3	7	0 (0.0; 0.0–35.4)	1 (14.3; 2.6–51.3)	0 (0.0; 0.0–35.4)	0 (0.0; 0.0–35.4)	1 (14.3; 2.6–51.3)
F4	8	0 (0.0; 0.0–32.4)	1 (12.5; 2.2–47.1)	0 (0.0; 0.0–32.4)	2 (25.0; 7.1–59.1)	4 (50.0; 21.5–78.5)
F5	8	0 (0.0; 0.0–32.4)	0 (0.0; 0.0–32.4)	0 (0.0; 0.0–32.4)	5 (62.5; 30.6–86.3)	1 (12.5; 2.2–47.1)
F6	9	1 (11.1; 2.0–43.5)	5 (55.6; 26.7–81.1)	2 (22.2; 6.3–54.7)	9 (100.0; 70.1–100.0)	3 (33.3; 12.1–64.6)
F7	14	0 (0.0; 0.0–21.5)	0 (0.0; 0.0–21.5)	0 (0.0; 0.0–21.5)	9 (64.3; 38.8–83.7)	2 (14.3; 4.0–39.9)
F8	10	2 (20.0; 5.7–51.0)	0 (0.0; 0.0–27.8)	3 (30.0; 10.8–60.3)	8 (80.0; 49.0–94.3)	6 (60.0; 31.3–83.2)
F9	10	1 (10.0; 1.8–40.4)	3 (30.0; 10.8–60.3)	0 (0.0; 0.0–27.8)	5 (50.0; 23.7–76.3)	5 (50.0; 23.7–76.3)
F10	8	2 (25.0; 7.1–59.1)	0 (0.0; 0.0–32.4)	1 (12.5; 2.2–47.1)	0 (0.0; 0.0–32.4)	0 (0.0; 0.0–32.4)
F11	13	3 (23.1; 8.2–50.3)	13 (100.0; 77.2–100.0)	10 (76.9; 49.7–91.8)	8 (61.5; 35.5–82.3)	1 (7.7; 1.4–33.3)
F12	6	0 (0.0; 0.0–39.0)	1 (16.7; 3.0–56.4)	4 (66.7; 30.0–90.3)	0 (0.0; 0.0–39.0)	0 (0.0; 0.0–39.0)
F13	9	0 (0.0; 0.0–29.9)	1 (11.1; 2.0–43.5)	6 (66.7; 35.4–87.9)	0 (0.0; 0.0–29.9)	1 (11.1; 2.0–43.5)
*P‐value*		*0.045*	*<0.001*	*<0.001*	*<0.001*	*0.004*

*Note*: *n* = number of pigs sampled from farm; 95% CI = 95% Wilson confidence interval; *Cryptosporidium* spp. exclude PCR dataset.

### 3.4 Intensity of Gastrointestinal Parasites in Pigs Using Complementary Coproscopic Techniques

The assessment of pig parasite load through complementary coproscopy methods indicates generally moderate to heavy GT parasite infections in the pigs. However, the parasite intensity, as measured by the number of egg/oocyst count per gram of faeces (EPG/OPG), was noticeably higher with the MM technique compared to the FECtechnique. This was particularly evident in the case of Trichuris spp., with a mean faecal egg count of 485.71 ± 389.13 EPG using the MM technique and 10.83 ± 13.87 EPG with the FECtechnique. Similarly, the mean EPG for strongyles was higher (2108.57 ± 4509.49) for the MM technique than the FECtechnique, as indicated in Table 5. Meanwhile, it is noteworthy that uniform intensity thresholds were used across parasite groups; however, the clinical impact of EPG/OPG varies substantially between taxa, and strongyle eggs were not identified to the genus. Therefore, intensity categories should be interpreted as descriptive measures of egg/oocyst shedding for epidemiological purposes, not as direct indicators of clinical disease. Data showed marked overdispersion, with high standard deviations indicating that a small proportion of heavily infected animals contributed disproportionately to total egg/oocyst output.

**TABLE 5:** Intensity of gastrointestinal (GIT) parasites identified in pigs using multiple coproscopic methods.

	Formol–ether concentration	Modified McMaster	Modified Ziehl–Neelsen
GIT parasite	Range (mean ± SD) eggs/gram	Range (mean ± SD) eggs or oocysts/gram	Range (mean ± SD) oocysts/gram
*Trichuris* spp.	1–51 (10.83 ± 13.87)^a^	100–1000 (485.71 ± 389.13)^b^	0
Strongyles	6–3672 (408.73 ± 839.90)^b^	100–25,500 (2108.57 ± 4509.49)^c^	0
*Balantioides* spp.	3–17268 (1477.17 ± 4332.56)^c^	0	0
*Eimeria* spp.	0	400–38200 (5381 ± 8989.22)^c^	0
*Cryptosporidium* spp.	0	0	2–2500 (186.26 ± 545.94)^a^

^a^ Light infection (<800 EPG/OPG).

^b^ Moderate infection (200–800 EPG/OPG).

^c^ Heavy infection (800+ EPG/OPG).

### 3.5 Agreement Between Coproscopy Methods

As shown in Table 6, both the FEC and MM techniques successfully detected *Trichuris* and strongylid eggs, although the FEC had a higher detection rate for *Trichuris* (63.2%), while the MM technique had a relatively higher detection rate for strongyles (57.4%). However, the FEC technique exclusively detected *Balantioides* spp. eggs, whereas the MM technique successfully detected *Eimeria* oocysts that were not detectable by the FEC. Additionally, *Cryptosporidium* oocysts were identified using the MZN technique, but not by the other coproscopy methods.

**TABLE 6:** Comparative parasite detection by formol–ether concentration (FEC), modified McMaster (MM), and modified Ziehl–Neelsen staining (MZN) techniques (*N* = 142).

Parasite	FEC positive	MM positive	MZN positive	Detected by one method	Detected by multiple methods	Combined microscopy positive
*Trichuris* spp.	12	7		11	4	15
*Strongyles*	26	35		29	0	29
*Balantioides* spp.	29	0		17	22	39
*Eimeria* spp.	0	49		49	0	49
*Cryptosporidium* spp.			31	31		31

Strongyles showed substantial agreement between FEC flotation and McMaster (*κ* = 0.647; 95% CI: 0.490−0.805), with McMaster identifying 13 additional positives over FEC (35 vs. 26; FEC sensitivity using McMaster as reference: 62.9%; McMaster sensitivity using FEC as reference: 84.6%), as shown in Table 7. Agreement for *Trichuris* spp. was fair (*κ* = 0.383; 95% CI: 0.074–0.662), with McMaster detecting only seven positives vs. 12 by FEC, reflecting its lower sensitivity for this organism (McMaster sensitivity: 33.3%; specificity: 97.7%). For *Balantioides* spp., the McMaster detected zero positives (vs. 29 by FEC); kappa could not be estimated owing to zero variance in the McMaster column. For *Eimeria* spp., FEC detected zero positives (vs. 49 by McMaster); kappa was similarly non‐estimable. Both represent substantive method‐failure findings rather than sampling artefacts.

**TABLE 7:** Agreement between formol–ether concentration and modified McMaster techniques for parasite detection in pigs (*N* = 142).

Parasite	FEC only	MM only	Both positive	Both negative	Cohen's *κ* (95% CI)	Interpretation
*Trichuris* spp.	8	3	4	15	0.383 (0.032‐0.733)	Fair
*Strongyles*	4	13	22	29	0.647 (0.490‐0.805)	Substantial
*Balantioides* spp.	29	0	0	39	Not estimated	MM detected no positives
*Eimeria* spp.	0	49	0	49	Not estimated	FEC detected no positives

*Note*: 0.00–0.20, slight; 0.21–0.40, fair; 0.41–0.60, moderate; 0.61–0.80, substantial; and 0.81–1.00, almost perfect agreement. “Not estimated” indicates that Cohen's *κ* could not be calculated because one method detected no positive samples, resulting in zero variance and preventing estimation of agreement; Both negative = samples negative by both methods. Cohen's *κ* (95% CI) was used to assess agreement between the two coproscopic techniques and was interpreted according to Landis and Koch (1977): <0.00, poor; Both positive = samples positive by both methods.

Abbreviations: CI, confidence interval. FEC only = samples positive by FEC only; FEC, formol–ether concentration; MM only = samples positive by MM only; MM, modified McMaster technique; *κ*, Cohen's kappa coefficient.

### 3.6 Detection of *Cryptosporidium* spp. by Modified Ziehl–Neelsen and PCR Techniques

Out of the subset of 92 samples analysed using nested PCR, 22 were *Cryptosporidium* oocyst‐positive, while 70 were oocyst‐negative. PCR amplification of the 18S rRNA gene detected *Cryptosporidium* spp. in 27 of the 92 (29.3%, 95% CI: 21.0−39.3) samples, improving parasite detection from 22 to 42 positives when PCR is combined with MZN (Table 8).

**TABLE 8:** Comparative *Cryptosporidium* detection by modified Ziehl–Neelsen staining (MZN) and PCR methods (*N* = 92).

Method	Positive	Prevalence (%)	95% CI
MZN	22	23.9	16.4–33.6
PCR	27	29.3	21.0–39.3
Combined	42	45.7	35.9–55.8

**FIGURE 1:**
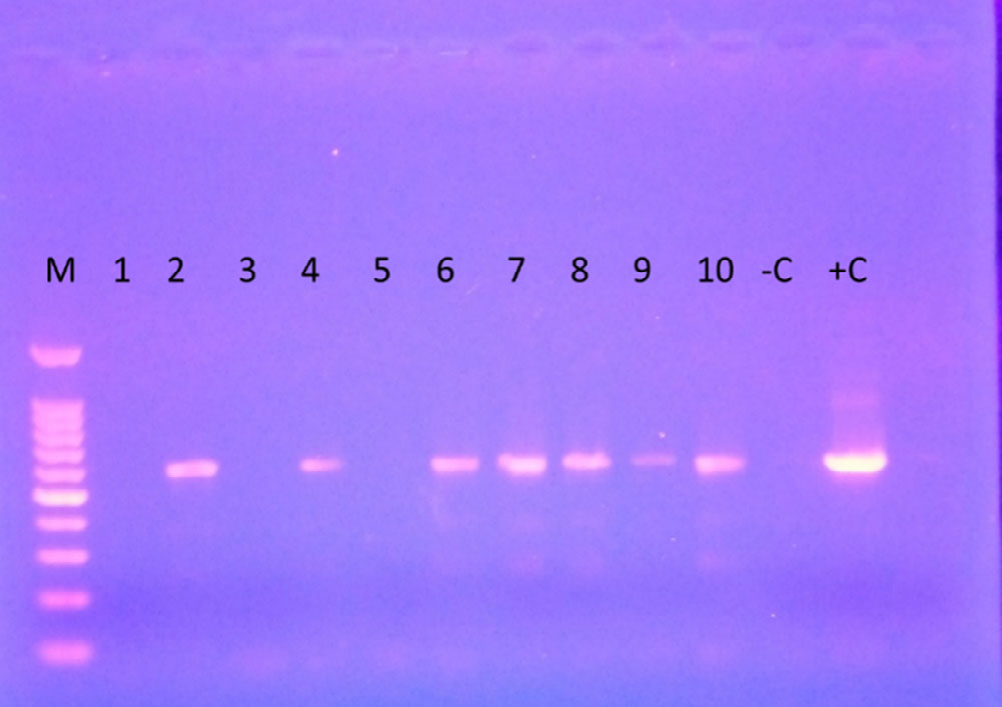
Agarose gel showing results of *Cryptosporidium* spp. positive pig‐stool samples. M: 100 bp DNA size marker. Positive samples: Lanes 2, 4, 6–10. Negative samples: Lanes 3 and 5. −C: negative control; +C: positive control.

Comparative analysis revealed that 7 pigs were positive by both PCR and MZN, 20 by PCR only, and 15 by MZN only, while 50 were negative (Table 9). *Cryptosporidium* showed slight agreement between MZN and PCR (*k* = 0.030; 95% CI 0.032−0.733), with PCR detecting 5 additional positives over MZN. A gel image of the PCR results for *Cryptosporidium* spp. is shown in Figure 1.

**TABLE 9:** Agreement between modified Ziehl–Neelsen staining and PCR methods techniques for *Cryptosporidium* detection in pigs (*N* = 92).

MZN only	PCR only	Both positive	Both negative	Cohen's *κ* (95% CI)	Interpretation
15	20	7	50	0.030 (0.032‐0.733)	Slight

*Note*: 0.00–0.20, slight; 0.21–0.40, fair; 0.41–0.60, moderate; 0.61–0.80, substantial; and 0.81–1.00, almost perfect agreement; Both negative = samples negative by both methods. Cohen's κ (95% CI) was used to assess agreement between the two techniques and was interpreted according to Landis and Koch (1977): <0.00, poor; Both positive = samples positive by both methods.

Abbreviations: CI, confidence interval. MZN only = samples positive by MZN only; MZN, modified Ziehl–Neelsen staining technique; PCR only = samples positive by PCR only; PCR, Polymerase chain reaction; *κ*, Cohen's kappa coefficient.

## 4 Discussion

The overall prevalence of gastrointestinal parasites in pigs in Kpone Katamanso in the Greater Accra Region of Ghana was 67.6% using complementary coproscopic techniques. Although this prevalence was lower than the 91.1% reported in Tolon and Kumbungu in northern Ghana and the 88.7% reported from the Ashanti Region (Addy et al. 2023; Osei et al. 2023), direct comparison should be interpreted cautiously because of differences in study populations, sampling strategies, diagnostic approaches, production systems, and agroecological conditions. Similarly, high prevalences have also been reported from neighbouring West African countries, including Nigeria, where gastrointestinal parasitism remains widespread in various pig production systems (Cyprian et al. 2022; Lekko et al. 2025). Collectively, these findings indicate that gastrointestinal parasitism continues to be an important health constraint in pig production across the region despite differences in management and environmental conditions.

Differences in parasite prevalence between this study and previous reports may reflect the combined influence of agroecological conditions, production systems, and farm management practices. Kpone Katamanso is located within Ghana's coastal savannah agroecological zone, whereas the studies by Osei et al. (2023) and Addy et al. (2023) were conducted in the deciduous forest and Guinea savannah zones, respectively. These agroecological zones differ in rainfall, temperature, humidity, and vegetation, all of which influence the survival, development, and transmission of parasite eggs, larvae, and oocysts in the environment (MOFA, 2016; Asare‐Nuamah and Botchway 2019; Bautista‐Garfias et al. 2022; Yadav and Upadhyay 2023). In addition, pig production systems differ across Ghana, ranging from extensive free‐range management to intensive confinement (Banson et al. 2014; Adjei et al. 2015). Although all the farms included in the present study operated intensive production systems, the contribution of management practices to the observed prevalence could not be evaluated because detailed information on biosecurity and anthelmintic use was incomplete. Consequently, the lower prevalence observed in this study should not be attributed solely to the production system. Nevertheless, the finding that all sampled farms had at least one infected pig indicates that gastrointestinal parasites remain widely distributed within the study area. Possible routes for parasite introduction and persistence include contaminated feed or water, manure accumulation, inadequate quarantine of replacement animals, contaminated equipment, and access of rodents or wild birds to pig houses. However, these pathways were not investigated in the present study and should therefore be regarded as plausible rather than demonstrated explanations.

The parasite profile observed in this study was broadly consistent with previous reports from Ghana, with *Eimeria* spp., *Cryptosporidium* spp., *Trichuris* spp., *Balantioides* spp., and strongyles being the predominant gastrointestinal parasites identified in pigs (Larbi et al. 2022; Addy et al. 2023; Osei et al. 2023; Otoo et al. 2026). Similar parasite assemblages have also been reported in pig populations from other sub‐Saharan African countries as well as other parts of the world, suggesting that these parasite groups remain widely distributed across diverse production systems despite differences in climate and husbandry practices (Abonyi and Njoga 2019; Bernard et al. 2021; Mramba and Massawe 2024; Thanasuwan et al. 2024; Villagómez‐Estrada et al. 2023). In contrast, *Ascaris suum*, which has been reported in pigs from other regions of Ghana (Addy et al. 2023; Osei et al. 2023), was not detected in the present study. Although intensive confinement may reduce exposure to contaminated soil, other factors such as previous anthelmintic treatment, improved sanitation, environmental conditions, or temporal variation in parasite transmission may also have contributed to its absence. Because information on deworming history and farm management was incomplete, the relative contribution of these factors could not be determined. The occurrence of mixed infections in some pigs further demonstrates that multiple gastrointestinal parasites may coexist within the same host, potentially increasing their cumulative effects on nutrient utilisation, growth, and overall productivity, although these production impacts were not evaluated in the present study.

*Cryptosporidium* spp. were detected using both MZN staining and nested PCR, confirming their occurrence among pigs sampled in Kpone Katamanso. The prevalence detected by MZN was lower than that reported by Larbi et al. (2022), although comparisons should be interpreted cautiously because of differences in study populations, production systems, and diagnostic approaches. Previous studies in Ghana have relied predominantly on conventional coproscopic techniques (Permin et al. 1999; Atawalna et al. 2016; Addy et al. 2023; Osei et al. 2023), whereas both Larbi et al. (2022) and the present study incorporated MZN specifically for *Cryptosporidium* detection. Within the subset analysed by both methods, nested PCR identified additional *Cryptosporidium*‐positive samples that were not detected by MZN. The slight agreement between the two methods (Cohen's *κ* = 0.030, 95% CI: 0.032−0.733) indicates limited concordance and suggests that each method detected infections missed by the other. Similar discordance between microscopy and PCR has been reported previously and may reflect biological factors, including intermittent oocyst shedding and low parasite burdens, together with methodological factors such as DNA quality and PCR inhibition (Sánchez et al., 2022). Because nested PCR was performed only for *Cryptosporidium* on a subset of samples and no diagnostic reference standard was available, these findings should be interpreted as demonstrating the complementary contribution of microscopy and molecular methods rather than comparative diagnostic performance.

Beyond *Cryptosporidium*, the comparison of FEC, MM, and MZN techniques showed that different coproscopic methods detected different parasite groups, with *Balantioides* spp. identified only by FEC, *Eimeria* spp. and *Cryptosporidium* spp. only by MM, and *Trichuris* spp. and strongyles detected by both FEC and MM. These findings demonstrate that the choice of diagnostic approach influences the parasite profile and prevalence estimates obtained in epidemiological surveys and support the use of complementary diagnostic methods where comprehensive parasite detection is required. Collectively, this study provides new epidemiological information on gastrointestinal parasites in pigs from Kpone Katamanso Municipality, an important peri‐urban pig production area where published data have been limited. However, because parasite identification was predominantly limited to the genus level and molecular characterisation was restricted to *Cryptosporidium*, the findings describe parasite occurrence rather than species distribution or zoonotic transmission. Future studies incorporating representative sampling, species‐level molecular characterisation, and risk‐factor analyses would strengthen understanding of gastrointestinal parasite epidemiology and support evidence‐based parasite surveillance and control in Ghana.

### 4.1 Limitations and Future Directions

The findings of this study should be interpreted within the context of the following limitations. First, the use of convenience and snowball sampling, necessitated by the absence of comprehensive census data for pig farms in Kpone Katamanso Municipality, limits the generalisability of the prevalence estimates beyond the sampled farms. Nevertheless, this approach enabled the inclusion of accessible pig farms within an important peri‐urban production area for which epidemiological information was previously unavailable.

Second, incomplete farm‐level management data precluded assessment of potential risk factors for gastrointestinal parasite occurrence, including the influence of husbandry practices and previous anthelmintic use. Consequently, the observed variation in parasite occurrence could not be linked to specific management or environmental factors.

Finally, parasite identification was based predominantly on complementary coproscopic techniques, while molecular confirmation was performed only for *Cryptosporidium* and did not include species or subtype characterisation. Accordingly, the study was designed to describe parasite occurrence and compare complementary diagnostic approaches under field conditions rather than determine species distribution, evaluate diagnostic accuracy, or infer zoonotic transmission.

Future studies incorporating probability‐based sampling, comprehensive farm‐level data, and species‐level molecular characterisation across the major gastrointestinal parasites would strengthen understanding of parasite epidemiology and support evidence‐based control strategies for pig production in Ghana.

## 5 Conclusion

This study demonstrates that gastrointestinal parasites remain an important health concern among pigs sampled from Kpone Katamanso Municipality despite the predominance of intensive production systems. The findings provide new epidemiological evidence from a peri‐urban pig production area of Ghana where published information has been limited and show that infected pigs were present on all participating farms. The complementary use of FEC, MM, and MZN techniques, together with targeted nested PCR for *Cryptosporidium*, showed that different diagnostic approaches identified overlapping but not identical infections, resulting in a more complete description of parasite occurrence under field conditions. Although species‐level identification was beyond the scope of this study, the findings establish an important baseline for future epidemiological investigations, molecular characterisation, and surveillance of gastrointestinal parasites in pigs. Collectively, these results contribute to the evidence base required to strengthen parasite surveillance and support the development of context‐appropriate control strategies for peri‐urban pig production systems in Ghana.

## Author Contributions

**Sylvia Afriyie Squire**: conceptualization, investigation, validation, methodology, visualization, formal analysis, project administration, supervision, resources, writing – original draft, data curation, software, writing – review and editing. **Linda Ama Owusuaa Amoah**: validation, formal analysis, writing – review and editing, writing – original draft, software, visualization, data curation. **Dennis Adusei**: conceptualization, methodology, supervision, formal analysis, validation, visualization, writing – original draft, data curation. **Doreen Dela Owusu‐Ntumy**: investigation, methodology, formal analysis, data curation, writing – original draft, writing – review and editing, validation, visualization, software, resources, conceptualization. **Antoinette V. Keleve**: conceptualization, investigation, methodology, validation, data curation, writing – original draft, formal analysis, project administration, resources, visualization, software, writing – review and editing.

## Funding

The authors have nothing to report.

## Ethics Statement

The authors confirm compliance with the journal's ethical policies. Ethical approval was obtained from the Animal Use and Care Committee (reference: CSIR050/2023). Faecal samples were collected following approved protocols, informed consent was obtained from pig owners, and all procedures adhered to accepted animal welfare standards.

## Conflicts of Interest

The authors declare no conflicts of interest.
